# Statistical Modeling of Agatston Score in Multi-Ethnic Study of Atherosclerosis (MESA)

**DOI:** 10.1371/journal.pone.0012036

**Published:** 2010-08-09

**Authors:** Shuangge Ma, Anna Liu, Jeffrey Carr, Wendy Post, Richard Kronmal

**Affiliations:** 1 School of Public Health, Yale University, New Haven, Connecticut, United States of America; 2 Department of Mathematics and Statistics, University of Massachusetts, Amherst, Massachusetts, United States of America; 3 Division of Radiological Sciences, Wake Forest University Health Sciences, Winston-Salem, North Carolina, United States of America; 4 Department of Cardiology, John Hopkins University, Baltimore, Maryland, United States of America; 5 Department of Biostatistics, University of Washington, Seattle, Washington, United States of America; University of Tor Vergata, Italy

## Abstract

The MESA (Multi-Ethnic Study of Atherosclerosis) is an ongoing study of the prevalence, risk factors, and progression of subclinical cardiovascular disease in a multi-ethnic cohort. It provides a valuable opportunity to examine the development and progression of CAC (coronary artery calcium), which is an important risk factor for the development of coronary heart disease. In MESA, about half of the CAC scores are zero and the rest are continuously distributed. Such data has been referred to as “zero-inflated data” and may be described using two-part models. Existing two-part model studies have limitations in that they usually consider parametric models only, make the assumption of known forms of the covariate effects, and focus only on the estimation property of the models. In this article, we investigate statistical modeling of CAC in MESA. Building on existing studies, we focus on two-part models. We investigate both parametric and semiparametric, and both proportional and nonproportional models. For various models, we study their estimation as well as prediction properties. We show that, to fully describe the relationship between covariates and CAC development, the semiparametric model with nonproportional covariate effects is needed. In contrast, for the purpose of prediction, the parametric model with proportional covariate effects is sufficient. This study provides a statistical basis for describing the behaviors of CAC and insights into its biological mechanisms.

## Introduction

**Figure 1:**
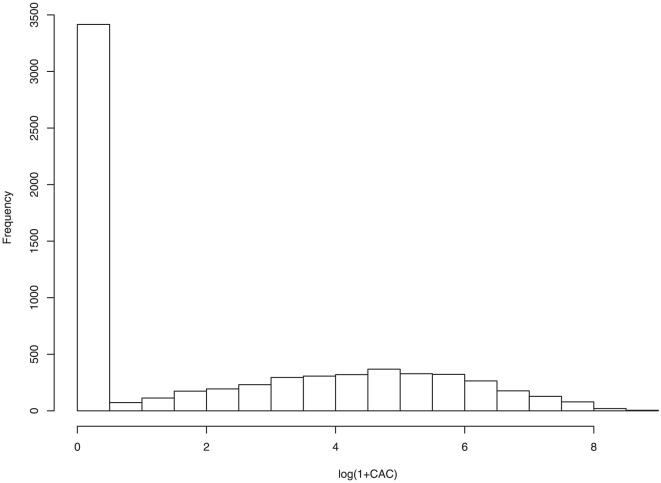
Histogram of log(1+CAC).

The MESA (Multi-Ethnic Study of Atherosclerosis) is an ongoing study of the prevalence, risk factors, and progression of subclinical cardiovascular disease in a multi-ethnic cohort (http://www.mesa-nhlbi.org/) [1]. It provides a valuable opportunity to investigate the development and progression of CAC (coronary artery calcium), which is an important risk factor for the development of coronary heart disease events [2]. In MESA, CAC is measured with the Agatston score, which is the amount of calcium at each lesion scaled by an attenuation factor and summed over all lesions [3]. The histogram of log(1+CAC) in Figure 1 shows that, about half of the CAC scores are zero and the rest are continuously distributed. In a relatively healthy cohort, such a mixture CAC distribution is commonly observed.

The CAC has a “point mass at zero+continuous” distribution and is a special case of zero-inflated data. Simple regression models are not capable of describing such data. It is not our intention to comprehensively review analytic methodologies for zero-inflated data. Instead, we focus on the statistical models for CAC. To describe nonzero CAC values, existing methods include generalized estimating equations [4], Tobit regression [5], zero-inflated normal model [6], quantile regression [7], and others. To describe zero versus nonzero CAC values, existing methods include logistic regression [5], relative risk regression, and others.

In MESA, after extensive comparisons and evaluations, Kronmal [8] suggested two-part models as the default for CAC. Two-part models have a long history in economic, statistical, and biomedical literature and can be a natural choice for data with a mixture distribution. With two-part models, the development of CAC is modeled in two steps (parts). The first step describes the development from zero to nonzero CAC values. In this step, the response variable is binary. The second step describes the progression of nonzero CAC values. In this step, the response variable is continuously distributed. The two steps have different purposes and different types of response variables, and hence demand different models with different covariate effects. Compared with other models that can also describe mixture data, two-part models have the advantage of being intuitive and not making strong assumptions on the unknown data generating mechanisms. On the negative side, our literature review suggests that existing two-part model studies may have the following limitations. First, they only consider parametric models. Such models are limited in that they cannot describe the subtle, nonlinear relationships between covariates and CAC. Second, they assume that the forms of the covariate effects are known. Such an assumption is usually not sufficiently justified. Third, they often focus on the estimation property and do not provide a comprehensive description of the models.

Building on existing studies [8], we investigate two-part CAC models in this article. This study has been motivated by the clinical importance of CAC and limitations of existing models. It advances from published studies in the following directions. First, besides parametric models, we also consider semiparametric models, with which we may discover the nonlinear relationships between covariates and CAC development. Second, multiple forms of covariate effects are considered. Particularly, besides ordinary nonproportional covariate effects, we also consider proportional covariate effects, which have fewer parameters, can be more accurately estimated, and may provide insights into the biological mechanisms underlying CAC development. Third, besides estimation, we also investigate the prediction performance of various models and thus are able to provide a more comprehensive description of those models.

## Methods

### The MESA Study

The MESA is a study of the characteristics of subclinical cardiovascular disease (disease detected non-invasively before it has produced clinical signs and symptoms) and the risk factors that predict progression to clinically overt cardiovascular disease or progression of the subclinical disease [1]. 6814 participants 45 to 84 years of age were recruited from six US communities from 2000 to 2002. Among them, 2619 are white, 1898 are African-American, 1494 are Hispanic, and 803 are Asian – predominantly Chinese descent. At recruitment, all participants were free of clinically apparent cardiovascular disease. Each participant received an extensive examination to determine coronary calcification, ventricular mass and function, flow-mediated endothelial vasodilation, carotid intimal-medial wall thickness and presence of atherosclerotic plaque, lower extremity vascular insufficiency, arterial wave forms, electrocardiographic (ECG) measures, standard coronary risk factors, sociodemographic factors, lifestyle factors, and psychosocial factors. Written consents were obtained from all participants.

CAC was measured with electron-beam computed tomography (EBT) at three field centers or multidetector computed tomography (MDCT) at the other three field centers. Each participant was scanned twice consecutively, and the results from the two scans were averaged to provide a more accurate estimation. The amount of calcium was quantified with the Agatston scoring method [3]. Calcium scores were adjusted with a standard calcium phantom that was scanned along with the participant. This phantom makes it possible to calibrate the degree of brightness between sites and participants. Rescan agreement was found to be high with both EBT and MDCT scanners. Interobserver agreement and intraobserver agreement were found to be satisfactory (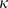 = 0.93 and 0.90, respectively) [9].

The MESA study has been approved by the Human Subjects Research Review Committee at University of Washington and all six sites. Detailed information is available at the MESA website http://www.mesa-nhlbi.org. Study presented in this article has been approved by the Human Subjects Research Review Committee at Yale University.

### Two-part CAC Models

The distribution of CAC is highly skewed. We make the logarithm transformation and study 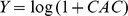. Figure 1 shows that, with probability ∼0.5, 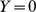. For 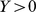, 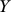 has a continuous distribution. Denote 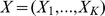 as the *K* covariates of interest.

Consider two-part models, where in the first part, we model the occurrence of a nonzero CAC value. More specifically, consider
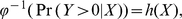
where 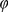 is the link function, 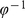 is the inverse of 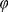, and 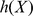 is the covariate effect. In the second part, consider
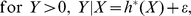
where 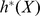 is the covariate effect, and 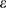 is the random error.

We determine the link function 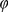 using the techniques described in [10] and find that the logistic link function, which has been suggested in [11], [12], is proper. We determine the distribution of random error using the approaches described in [13] and find that the normal distribution is proper. This is intuitively reasonable by “eyeballing” Figure 1. There are multiple possibilities for the covariate effects, including:

- Parametric, proportional covariate effects:
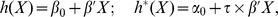
where 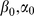 are the unknown intercepts, 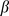 is the unknown length-*K* regression coefficients, and 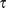 is the unknown scale parameter;
- Parametric, nonproportional covariate effects:
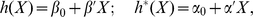
where 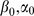 are the unknown intercepts, 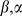 are the unknown length-*K* regression coefficients, and there is no proportionality constraint on 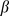 and 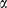;
- Semiparametric, proportional covariate effects:
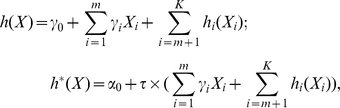
where 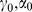 are the unknown intercepts, 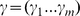 is the length-*m* unknown regression coefficients, 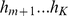 are the *K-m* unknown nonparametric covariate effects, and 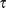 is the unknown scale parameter;
- Semiparametric, nonproportional covariate effects:
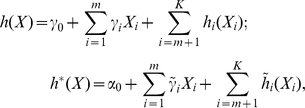
where 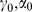 are the unknown intercepts, 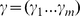 and 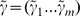 are the length-*m* unknown regression coefficients, 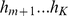, 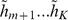 are the unknown nonparametric covariate effects, and there is no proportionality constraint on 
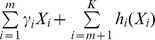
 and 
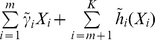
.

#### Remarks: Parametric and semiparametric models

Models(i) and (ii) are parametric, whereas models (iii) and (iv) are semiparametric. There is a rich literature on the advantages and disadvantages of parametric and semiparametric models [14]. Parametric models assume linear relationships between covariates (or their transformations) and response variables. They are usually easy to interpret, with the regression coefficients measuring the increase rates of response variables with changes of covariates. In addition, they can be easily estimated using many existing software and the estimates usually have the desired root-n convergence rate. Statistical inference can be easily conducted using likelihood-based methods. On the negative side, the assumption of linear relationships can be limited and subject to model misspecification. Semiparametric models, on the other hand, allow nonlinear relationships between covariates and response variables. Thus, they are able to describe more subtle data structure. The tradeoff is that semiparametric models can be hard to estimate and interpret. In addition, the estimates of nonparametric functions may not have the desired root-n convergence rate. Moreover, inference with semiparametric models may not be straightforward. Computationally intensive methods, such as the bootstrap or jackknife, may be needed. Of note, most existing studies assume parametric two-part models. In this study, to comprehensively describe CAC, both parametric and semiparametric models are considered.

#### Remarks: Proportional and nonproportional models

Most existing two-part models share a similar spirit with models (ii) and (iv) in that there is no constraint on the covariate effects 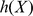 and 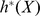. Unlike those models, models (i) and (iii) have a proportionality constraint. That is, other than the intercepts, the covariate effects 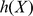 and 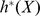 differ only by a scale parameter. Compared with nonproportional models, proportional models have fewer unknown parameters and thus can be more accurately estimated. This improved accuracy has been rigorously proved and observed in numerical studies [11]. In addition, in proportional models, covariates contribute to 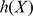 and 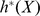 in the same manner. Thus, it is reasonable to hypothesize that, when the proportionality holds, the same biological process determines whether the CAC is zero as well as its actual value if nonzero. This may provide insights into the biological mechanisms underlying CAC development. Moreover, under proportionality, the same index 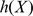 can be used to predict the whole range of CAC – from zero to nonzero as well as progression of nonzero values. This may simplify practice involving predicting the CAC values.

#### Remarks: Estimation and prediction

With a statistical model, we are interested in its two closely related but distinct properties. The first is the estimation property, where the goal is to fully describe the relationship between covariates and response variable. The second is the prediction property, where the goal is to accurately predict values of the response variable for subjects that are not used in model building. Theoretically speaking, there exists a true data generating model. This model not only provides the best description of the relationship between covariates and response but also has the best prediction performance. However, in practice with finite sample data, the true model is not known, and the models most suitable for estimation and prediction may differ.

With a simple linear regression model (M1): 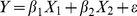, we consider scenarios under which the models most suitable for estimation and prediction are different and possible causes of the difference. It is expected that similar arguments hold for more complicated models. We have conducted a small scale simulation, where we fix the values of 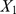 and 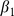. With the simulated data, we are able to increase the magnitudes of 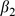 and 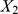, but keep 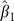 (the estimate of 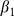) statistically significant with p-value<0.05 (more details available upon request).

We also consider the alternative model (M2): 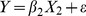. For estimation, since the goal is to fully describe the relationship between the covariates and response variable, model (M1) is needed, while model (M2) is misspecified and improper. For prediction, the goal is to minimize the squared error *SE = (predicted value - observed value)^2^* for subjects not used in model building. This quantity can be decomposed into two components. The first is a bias component, and the second is a variance component. Model (M2) is misspecified, so it may have the bias component larger than that of (M1). However, model (M2) has fewer unknown parameters and can be more accurately estimated. So the variance component for (M2) may be smaller than that for (M1). Thus, because of the bias-variance tradeoff, the misspecified (M2) can be more suitable for prediction. In studies of statistical models for CAC, the two aspects of model fitting have not been well distinguished, and the best estimation models have been used for prediction without rigorous justification, or vice versa. Our study shows that, for CAC in MESA, the models most suitable for estimation and prediction are in fact different.

### Estimation and inference methods

With a normally distributed random error, up to a constant, the log-likelihood function for a single observation is
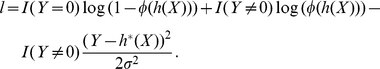
Assume *n* iid observations. Denote 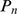 as the empirical measure.

With models (i) and (ii), we consider the maximum likelihood estimates (MLE), which are defined as the maximizers of 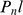. Under regularity conditions, the MLEs are 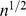 consistent and asymptotically normally distributed. This result can be established using the standard M-estimation theories.

With model (iii), we further assume that 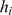
s are smooth functions (or more specifically, spline functions). This assumption has been motivated by the observation that the change of covariate values affects CAC development in a continuous manner. Following [15], we consider the penalized maximum likelihood estimate (PMLE) defined as the maximizer of 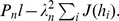 Here, 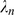 is the data-dependent tuning parameter and can be selected using the approach described in [12], [16]. 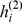 is the second-order derivative of 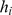, and 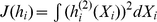 is the penalty on smoothness [15]. We assume that, (A1) 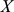 belongs to a compact subset of 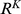; 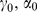 and 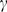 are bounded; (A2) The asymptotic variance matrix of the parametric parameters is non-singular and component-wise bounded; and (A3) 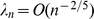. Under (A1)–(A3), the PMLEs of 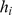
s are 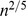 consistent, and the PMLEs of the parametric parameters are 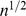 consistent and asymptotically normally distributed. This result can be proved using the empirical processes techniques described in [17].

With model (iv), we adopt a similar estimation strategy and consider the PMLE defined as the maximizer of 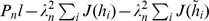. Under conditions similar to (A1)–(A3), the PMLEs of the nonparametric parameters are 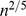 consistent, and the PMLEs of the parametric parameters are 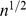 consistent and asymptotically normally distributed.

With parametric models (i) and (ii), inference can be based on the asymptotic normality result and the Fisher information matrix. However, with semiparametric models (iii) and (iv), such an approach involves smoothed estimation and is very difficult to employ. We propose the following bootstrap approach for inference of all parameters in all models: (a) Fit the model and compute the MLEs (PMLEs); (b) With the observed covariate values, generate random errors from the normal distribution with mean zero and variance 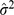; (c) Generate the binary indicators 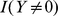 using the logistic model. For those with 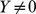, generate the continuous 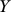 values; (d) With the generated responses, re-estimate the model; (e) Repeat steps (a)–(d) B (for example 500) times. We estimate the variances of the MLEs (PMLEs) using the variances of estimates generated using the bootstrap samples.

## Results

### Estimation Properties

We collect measurements on the following covariates, which have been suggested as possibly associated with CAC in various publications: gender (female is used as the reference group), race/ethnicity (Caucasian, Chinese, African-American, and Hispanic; Caucasian is used as the reference group), former smoker (binary indicator), current smoker (binary indicator), diabetes (binary indicator), SBP (systolic blood pressure), DBP (diastolic blood pressure), age, BMI (body mass index), LDL cholesterol, and HDL cholesterol. We consider the following parametric models: (i.1) model (i) with linear effects for all covariates; (i.2) model (i) with linear effects for all covariates plus quadratic effects for LDL and HDL; (ii.1) model (ii) with linear effects for all covariates; and (ii.2) model (ii) with linear effects for all covariates plus quadratic effects for LDL and HDL. Models (i.1) and (ii.1) are more commonly adopted in practice, whereas models (i.2) and (ii.2) have been motivated by the nonproportional semiparametric model, i.e, the “biggest model”, and suggested by a reviewer. In semiparametric models (iii) and (iv), among the 13 covariates, 7 are binary, which naturally have parametric effects. Our preliminary analysis also suggests parametric covariate effects for SBP and DBP. Thus, there are 9 parametric covariate effects and 4 nonparametric ones. There are a total of six models considered.

**Figure 2:**
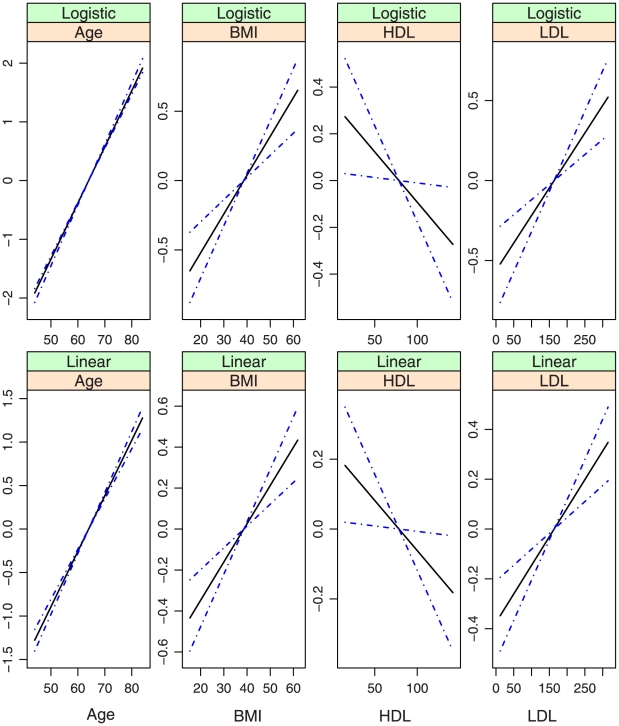
Model (i.1): estimated covariate effects for age, BMI, HDL, and LDL. The solid line is the estimate. The dash-dotted lines are the point-wise 95% confidence intervals. The y-axis is the value of the function.

**Figure 3:**
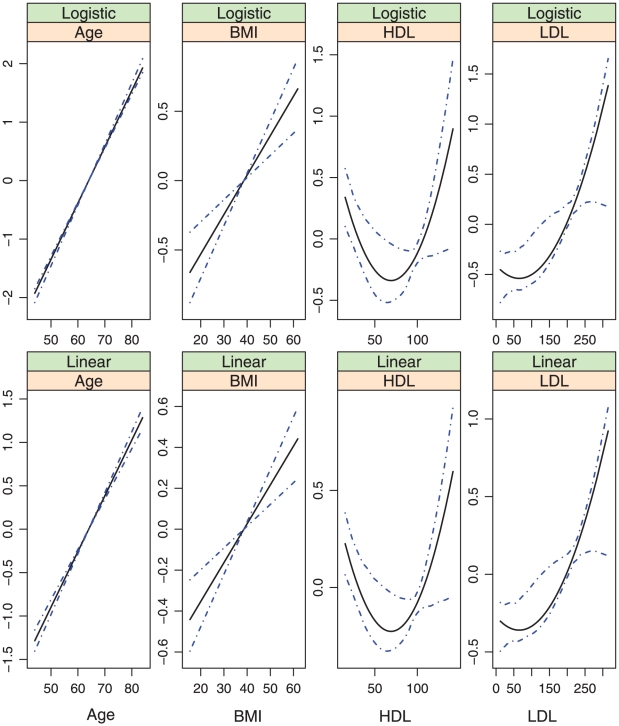
Model (i.2): estimated covariate effects for age, BMI, HDL, and LDL. The solid line is the estimate. The dash-dotted lines are the point-wise 95% confidence intervals. The y-axis is the value of the function.

**Figure 4:**
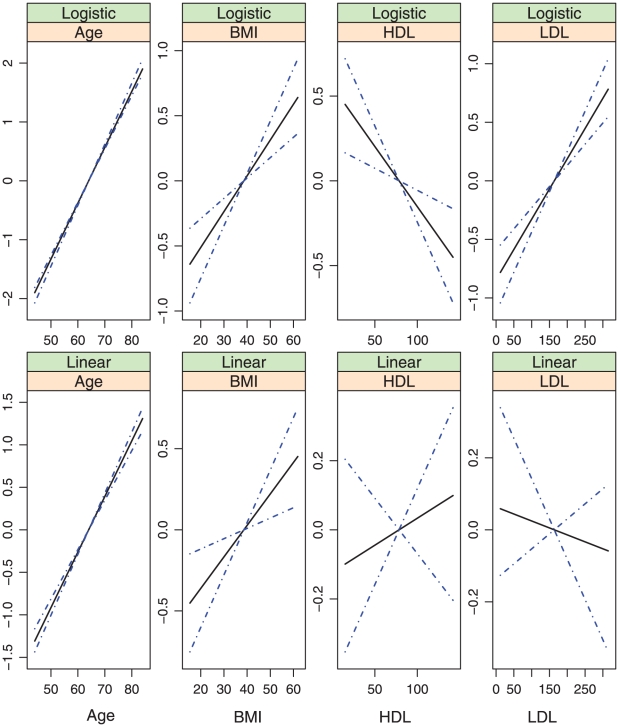
Model (ii.1): estimated covariate effects for age, BMI, HDL, and LDL. The solid line is the estimate. The dash-dotted lines are the point-wise 95% confidence intervals. The y-axis is the value of the function.

**Figure 5:**
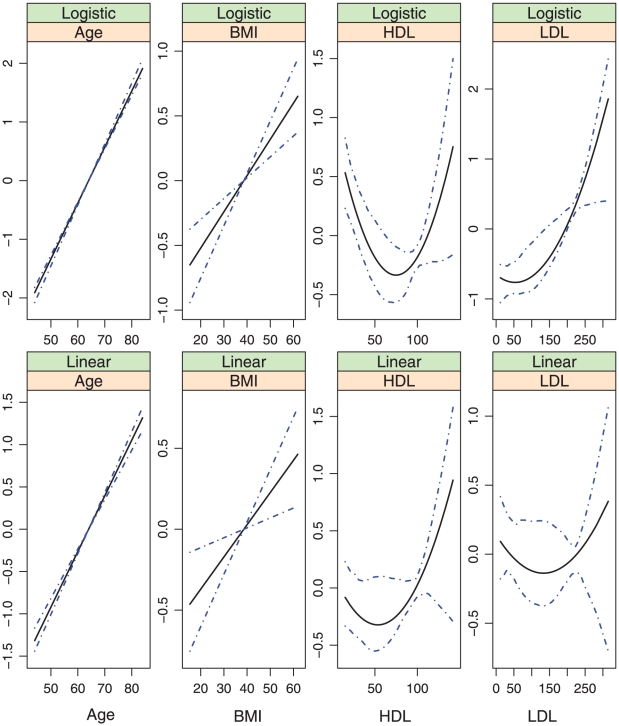
Model (ii.2): estimated covariate effects for age, BMI, HDL, and LDL. The solid line is the estimate. The dash-dotted lines are the point-wise 95% confidence intervals. The y-axis is the value of the function.

**Figure 6:**
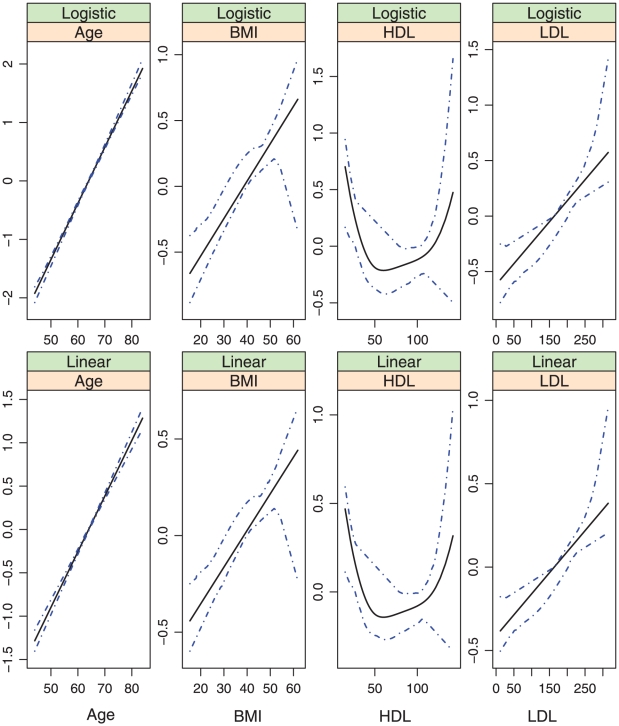
Model (iii): estimated covariate effects for age, BMI, HDL, and LDL. The solid line is the estimate. The dash-dotted lines are the point-wise 95% confidence intervals. The y-axis is the value of the function.

**Figure 7:**
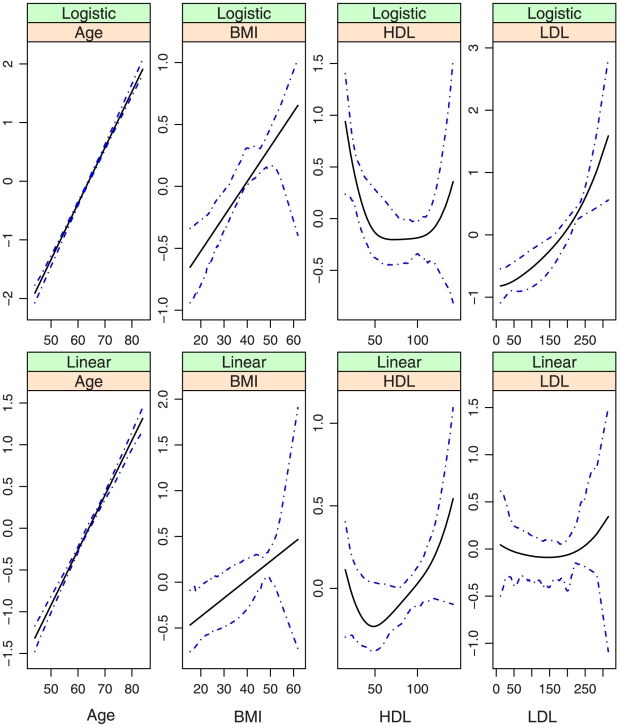
Model (iv): estimated covariate effects for age, BMI, HDL, and LDL. The solid line is the estimate. The dash-dotted lines are the point-wise 95% confidence intervals. The y-axis is the value of the function.

For 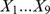, covariates with parametric effects in all models, we show the MLEs (PMLEs) in Table 1. For all covariates, their estimates under different models have almost the same signs. Thus the biological conclusions on whether they are positively or negatively associated with CAC are the same in all models. However, the magnitudes of the estimates may be considerably different. For example, the estimates of the regression coefficients for 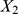 in the linear parts are −0.151, −0.144, −0.291, −0.273, −0.141, and −0.285, respectively. For 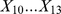, covariates with nonparametric effects in models (iii) and (iv), we show the estimates and point-wise 95% confidence intervals in Figures 2–7. We note that the lines intercept at the mean of X-axis since every fitted line has been mean-centered for identifiability. In addition, estimates under models (i) and (ii) are straight lines (i.e, parametric). Figures 2–7 show that, the estimates of the Age and BMI effects under different models are reasonably close. It is interesting that under models (iii) and (iv), the nonparametric estimates of the Age and BMI effects are close to linear functions. However, the estimates of the HDL and LDL effects under different models are significantly different, with the HDL effect in models (i.2), (ii.2), (iii) and (iv) and the LDL effect in models (i.2), (ii.2) and (iv) significantly deviating from straight lines.

**Table 1:** Estimated regression coefficients (standard errors) for covariates with parametric effects in all models.

Covariate	Model (i.1)		Model (i.2)		Model (ii.1)		Model (ii.2)		Model (iii)		Model (iv)	
	Logistic	Linear	Logistic	Linear	Logistic	Linear	Logistic	Linear	Logistic	Linear	Logistic	Linear
Gender:Male( 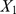 )	0.988(0.063)	0.659(0.042)	0.971(0.063)	0.646(0.047)	0.966(0.072)	0.688(0.085)	0.951(0.073)	0.675(0.075)	0.969(0.062)	0.647(0.047)	0.945(0.074)	0.681(0.073)
Race:Chinese ( 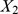 )	−0.227(0.078)	−0.151(0.047)	−0.217(0.078)	−0.144(0.052)	−0.140(0.092)	−0.291(0.074)	−0.134(0.092)	−0.273(0.099)	−0.211(0.079)	−0.141(0.053)	−0.119(0.092)	−0.285(0.099)
Race: AfricanAmerican ( 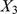 )	−0.736(0.065)	−0.491(0.052)	−0.756(0.065)	−0.503(0.046)	−0.794(0.070)	−0.384(0.100)	−0.810(0.071)	−0.406(0.080)	−0.731(0.066)	−0.488(0.047)	−0.787(0.070)	−0.398(0.081)
Race:Hispanic ( 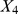 )	−0.593(0.065)	−0.396(0.046)	−0.604(0.064)	−0.402(0.042)	−0.626(0.073)	−0.348(0.081)	−0.636(0.074)	−0.354(0.084)	−0.596(0.067)	−0.398(0.044)	−0.628(0.071)	−0.358(0.085)
Former smoker( 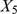 )	0.352(0.059)	0.234(0.038)	0.354(0.059)	0.235(0.038)	0.368(0.069)	0.211(0.069)	0.366(0.069)	0.218(0.069)	0.354(0.060)	0.236(0.039)	0.370(0.072)	0.213(0.071)
Current smoker( 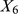 )	0.580(0.080)	0.387(0.054)	0.579(0.080)	0.385(0.054)	0.620(0.091)	0.329(0.101)	0.617(0.091)	0.328(0.101)	0.573(0.083)	0.382(0.056)	0.609(0.094)	0.328(0.096)
Diabetes ( 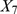 )	0.309(0.058)	0.206(0.041)	0.308(0.057)	0.205(0.041)	0.255(0.071)	0.281(0.066)	0.253(0.070)	0.281(0.066)	0.300(0.057)	0.201(0.041)	0.243(0.070)	0.275(0.068)
SBP ( 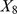 )	0.008(0.002)	0.005(0.001)	0.008(0.002)	0.005(0.001)	0.009(0.002)	0.004(0.002)	0.009(0.002)	0.004(0.002)	0.008(0.002)	0.005(0.001)	0.009(0.002)	0.004(0.002)
DBP ( 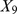 )	−0.0009(0.004)	−0.0006 (0.002)	−0.001(0.004)	−0.0006 (0.002)	−0.003(0.004)	0.003(0.004)	−0.003(0.004)	0.002(0.004)	−0.001 (0.004)	−0.0007 (0.002)	−0.0034 (0.004)	0.0032(0.004)

Among the six models, models (i.1)-(iii) are special cases of model (iv). Table 1 and Figures 2–7 show that, the estimates under model (iv) are considerably different from their counterparts under models (i)–(iii). To further quantify whether the differences between the estimates under model (iv) and those under the other five models are significant, we consider the following bootstrap-based likelihood ratio tests [16]. Consider, for example, the test


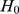
: *model (iv) can be simplified as (i.1)* versus 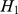: *model (iv) cannot be simplified*.

We consider the likelihood ratio test statistic 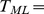 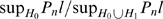. Hypothesis testing using the bootstrap approach consists of the following steps: (a) Fit the Null model; (b) With the observed covariates, generate the random errors and 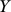 values; (c) With the generated responses, estimate the model. Compute the test statistic 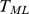; (d) Repeat steps (a)–(c) B (for example 500) times. An empirical p-value can be computed. We can see that, this procedure is a byproduct of the inference procedure and does not incur any additional computational cost. We conduct hypothesis testing and find that all the five comparisons are significant with the Bonferroni adjustment for multiple comparisons.

Based on the above results, we conclude that, *to fully describe the relationship between CAC and risk factors*, *the semiparametric nonproportional model (iv) is needed*. Figure 7 shows that, (a) the nonparametric age and BMI effects are close to their counterparts under the alternative models and may be simplified to parametric effects; (b) In both the logistic and the linear parts of the model, the HDL effects are highly nonlinear and have a “U” shape. When the values of the other covariates are fixed, a moderate value of HDL corresponds to the smallest probability of nonzero CAC as well as the smallest value of CAC if nonzero. This study is among the first to identify such an interesting relationship between HDL and CAC. The biological implications of this finding are not clear and need further investigation; and (c) The LDL effects also deviate significantly from linear in both parts of the model. More specifically, it also has a “U” shape in the linear part. However, the magnitude is very small. The LDL effect is monotone, increasing in the logistic part, suggesting that a higher level of LDL is associated with a higher probability of nonzero CAC. This finding is consistent with the literature.

### Prediction Properties

To evaluate the prediction performance, ideally, two independent datasets (one training set and one testing set) from studies with comparable designs are needed. We are not able to find a study fully comparable to MESA. As an alternative, we consider the following Monte Carlo-based approach. (a) Randomly split the data in to a training set and a testing set with equal sizes; (b) Estimate the unknown parameters using the training set only; (c) Make predictions for subjects in the testing set. Specifically, for a subject, first predict the probability of a nonzero CAC using the logistic regression model. Dichotomize the predicted probability at 0.5 and create the binary CAC status (zero or nonzero). If a nonzero CAC status is obtained, predict its actual value using the linear regression model; and (d) To avoid bias caused by an extreme split, repeat Steps (a)–(c) 500 times. Compute summary statistics.

In Step 1, we use random partition to generate independent training and testing sets. To avoid an extreme partition, multiple partitions are carried out. In the prediction evaluation, we are interested in the probability of correctly predicting the binary CAC status (zero or nonzero) as well as the overall mean squared error (MSE), which measures the ability to predict the actual CAC values. Under the six models, the mean error rates for predicting zero versus nonzero CAC are
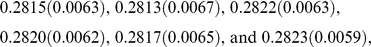
where numbers in the “()” are the standard deviations. The overall MSEs are
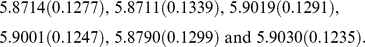



The above results suggest that, despite their significantly different estimation results, all models have similar prediction performance. It is interesting to note that model (i.2), which has parametric proportional covariate effects, has prediction performance better than all of the other models (although the differences are small). As model (i.2) is a submodel of model (iv), this finding may seem counterintuitive. However, as discussed above, it can be explained by the bias-variance tradeoff. Although the prediction performance of model (i.2) is only slightly better than that of the other models, it has fewer unknown parameters than four of the alternative models and is easy to estimate. Thus, we conclude that model (i.2) is the most suitable for the purpose of prediction.

## Discussion

In the above sections, interesting findings include the nonlinear relationships found in model (iv) and the discrepancy between the models most suitable for estimation and prediction. Examination of Table 1 and Figures 3 and 7 suggests that, the estimates of the following covariate effects differ considerably between models (i.2) and (iv): Race:Chinese, DBP, HDL, and LDL. Among them, the Race:Chinese and DBP effects are parametric. Under the two models, the magnitudes of their estimates differ. However, the signs are almost the same, suggesting similar qualitative conclusions. The HDL and LDL effects are nonparametric, and even the qualitative conclusions are different in the two models. For example, in the logistic parts, the right end of HDL has a larger effect in model (i.2), whereas the left end has a larger effect in model (iv).

As discussed in Introduction, in the asymptotic sense when the sample size goes to infinity, one single model should be the most suitable for estimation and prediction. That is, the discrepancy we observe should disappear. However, any practical data has a finite sample size. The MESA has a sample size of 6814, which is larger than that of many studies. Thus, it may be reasonable to expect similar discrepancy in other studies.

Models (i) and (iii) assume that the two covariate effects are perfectly proportional, whereas models (ii) and (iv) assume no proportionality. There are intermediate, partially proportional models with some covariate effects being proportional and the others not. Such models are as difficult to interpret and estimate as nonproportional models and hence not pursued. Figure 7 suggests that it may be possible to model the Age and BMI effects using parametric functions. However, such simplification cannot change the semiparametric, nonproportional nature of model (iv) and is not pursued. Another possible prediction accuracy measurement is the mean squared error for the positive CAC values only. However, the set of subjects with predicted positive CAC values is different from the set observed. The MSE for positive CAC values has ambiguity in its definition and is not pursued.

Our conclusions on the CAC models are based on the analysis of MESA. There are a few other studies examining similar cardiovascular problems, including the CHICAGO study [18], the UIC database [19], the CARDIA study [20], and others. It is of interest to analyze those datasets and examine if the results obtained in this study hold in general. Such an endeavor will require access to several non-public databases. In MESA, CAC is measured with the Agatston score, which may not provide a full description of coronary calcification. To comprehensively understand coronary calcification, other measurements may need to be considered.

Coronary artery calcification is an important predictor of cardiovascular disease events. Our literature review suggests certain limitations of existing CAC modeling studies. In this article, we analyze the MESA data and systematically investigate various CAC models. Building on existing studies including [8], [11], we focus on two-part models, which are able to describe data with a mixture distribution while being intuitive and easy to implement. With the link function and distribution of random error determined using existing techniques, we focus on the covariate effects. Particularly, for both parametric and semiparametric, both proportional and nonproportional models, we investigate the estimation and prediction properties. We find that, to fully describe the relationship between CAC and risk factors, model (iv) is the most suitable. However, for predicting the response variable for subjects not used in estimation, model (i.2) is the most suitable. The discrepancy between estimation and prediction models has not been well discussed in the CAC literature. This study may be the first step in understanding that. Although we focus on the CAC models in MESA, the proposed models and analysis methodologies may have broader applications. In addition, we conjecture that the conclusion may also hold for other cardiovascular disease measurements and other datasets.
